# 6-Meth­oxy-1,3-benzothia­zol-2-amine

**DOI:** 10.1107/S1600536812028152

**Published:** 2012-06-30

**Authors:** Aamer Saeed, Hummera Rafique, Ulrich Flörke

**Affiliations:** a Department of Chemistry, Quaid-i-Azam University, Islamabad 45320, Pakistan; b Department Chemie, Fakultät fur Naturwissenschaften, Universität Paderborn, Warburgerstrasse 100, D-33098 Paderborn, Germany.

## Abstract

The title compound, C_8_H_8_N_2_OS, is almost planar, the C—C—O—C torsion angle associated with the meth­oxy group being 0.72 (1)°. Inter­molecular amine N—H⋯N hydrogen-bonding inter­actions form inversion dimers [graph set *R*_2_^2^(8)] which are extended into chains along the *b* axis through amine N—H⋯O hydrogen bonds.

## Related literature

For information on various important biological activities of amino­benzothia­zoles, see: Hutchinson *et al.* (2002 ▶); Benavides *et al.* (1985 ▶); La’cova *et al.* (1991 ▶). For their pharmaceutical applications, see: Suter & Zutter (1967 ▶); Sawhney *et al.* (1978 ▶); Bensimon *et al.* (1994 ▶); Foscolos *et al.* (1977 ▶); Shirke *et al.* (1990 ▶); Paget *et al.* (1969 ▶); Domino *et al.* (1952 ▶). For anti­microbial and pesticidal activities, see: Pattan *et al.* (2002 ▶); Kaufmann (1935 ▶). For related structures see: Saeed *et al.* (2007 ▶); Sun *et al.* (2011 ▶). For graph-set analysis, see: Etter *et al.* (1990 ▶).
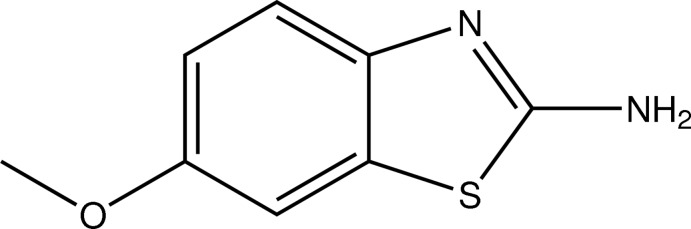


## Experimental

### 

#### Crystal data

C_8_H_8_N_2_OS
*M* *_r_* = 180.23
Orthorhombic, 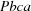

*a* = 15.060 (2) Å
*b* = 6.6997 (11) Å
*c* = 16.649 (3) Å
*V* = 1679.8 (5) Å^3^
*Z* = 8
Mo *K*α radiation
μ = 0.33 mm^−1^
*T* = 130 K
0.47 × 0.23 × 0.14 mm

#### Data collection

Bruker SMART APEX CCD diffractometer
Absorption correction: multi-scan (*SADABS*; Sheldrick, 2004 ▶) *T*_min_ = 0.859, *T*_max_ = 0.955
14745 measured reflections
2010 independent reflections
1771 reflections with *I* > 2σ(*I*)
*R*_int_ = 0.029

#### Refinement

*R*[*F* ^2^ > 2σ(*F* ^2^)] = 0.033
*wR*(*F* ^2^) = 0.094
*S* = 1.06
2010 reflections
110 parameters
H-atom parameters constrained
Δρ_max_ = 0.40 e Å^−3^
Δρ_min_ = −0.20 e Å^−3^

### 

Data collection: *SMART* (Bruker, 2002 ▶); cell refinement: *SAINT* (Bruker, 2002 ▶); data reduction: *SAINT*; program(s) used to solve structure: *SHELXS97* (Sheldrick, 2008 ▶); program(s) used to refine structure: *SHELXL97* (Sheldrick, 2008 ▶); molecular graphics: *SHELXTL* (Sheldrick, 2008 ▶); software used to prepare material for publication: *SHELXTL* and local programs.

## Supplementary Material

Crystal structure: contains datablock(s) global, I. DOI: 10.1107/S1600536812028152/zs2217sup1.cif

Structure factors: contains datablock(s) I. DOI: 10.1107/S1600536812028152/zs2217Isup2.hkl

Supplementary material file. DOI: 10.1107/S1600536812028152/zs2217Isup3.mol

Supplementary material file. DOI: 10.1107/S1600536812028152/zs2217Isup4.cml

Additional supplementary materials:  crystallographic information; 3D view; checkCIF report

## Figures and Tables

**Table 1:** Hydrogen-bond geometry (Å, °)

*D*—H⋯*A*	*D*—H	H⋯*A*	*D*⋯*A*	*D*—H⋯*A*
N2—H2*A*⋯N1^i^	0.88	2.13	2.9774 (16)	163
N2—H2*B*⋯O1^ii^	0.88	2.10	2.9655 (16)	170

Symmetry codes: (i) 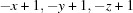; (ii) 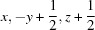.

**Fig. 1.**
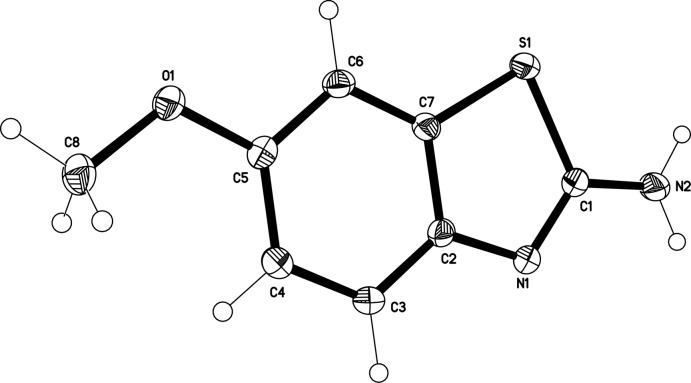
Molecular structure of title compound showing atom numbering scheme and displacement ellipsoids drawn at the 50% probability level.

**Fig. 2.**
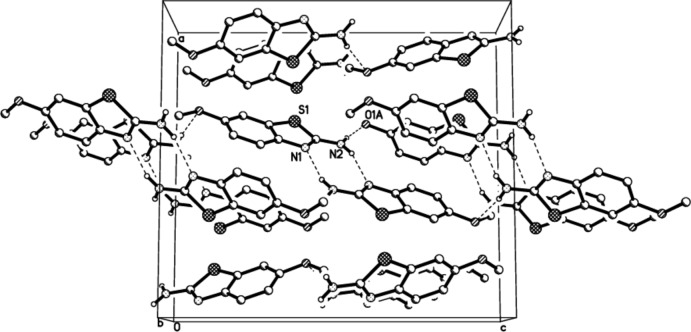
Crystal packing viewed along (010) with intermolecular hydrogen bonds indicated as dashed lines. H-atoms not involved in hydrogen bonding are omitted.

## supplementary crystallographic information

### Comment

2-Aminobenzothiazoles display a broad spectrum of important pharmaceutical applications and a number of derivatives are therapeutic agents for the treatment of various diseases e.g. diabetes (Suter & Zutter, 1967); inflammation (Sawhney *et al.*, 1978); amyotrophic lateral sclerosis (Bensimon *et al.*, 1994); analgesia (Foscolos *et al.*, 1977); tuberculosis (Shirke *et al.* 1990); viral infections (Paget *et al.*, 1969), and as central muscle relaxants (Domino *et al.*, 1952). Riluzole [6-(trifluoromethoxy)-2-benzothiazolamine] possesses potent anticonvulsant and neuroprotective effects (Benavides *et al.*, 1985). 6-Nitro or 6-amino 2-substituted benzothiazoles and fluorobenzothiazoles possess significant antimicrobial activity (Pattan *et al.*, 2002) and 6-ethoxy-2-amino benzothiazole is a strong local anesthetic agent (La'cova *et al.*, 1991).

The title compound, C_8_H_8_N_2_OS, is almost planar (Fig. 1) with the C4—C5—O1—C8 torsion angle associated with the methoxy group = 0.72 (1)° and the amine hydrogen atoms lying in the molecular plane. Intermolecular amine N—H···N hydrogen-bonding interactions (Table 1) form centrosymmetric cyclic dimers [graph set *R*^2^_2_(8): Etter *et al.*, 1990] which are extended into one-dimensional chains along the *b* axis, through amine N—H···O hydrogen bonds (Fig. 2).

### Experimental

A mixture of *p*-anisidine (3.7 g, 0.03 mol) and potassium thiocyanate (11.6 g, 0.12 mol) in AcOH (45 ml) was stirred at 20 °C for 10 minutes. A solution of bromine (1.5 ml, 0.03 mol) in AcOH (20 ml) was added over 20 min and the reaction mixture was stirred for 21 h at room temperature. The reaction mixture was poured into cold NH4OH (90 ml) and extracted with EtOAc. The organic phase was washed with water, dried, filtered and evaporated. The crude product obtained was recrystallized using ethanol as solvent. Yield = 83%; m.p. = 145-147 °C; IR (KBr) 3389 (NH), 2733 (C-S), 1644 (C=N), 1585 (C=C), 1442 (C-N) cm-1; 1H NMR (CDCl3, ? p.p.m.): 7.47 (1*H*, d, J = 8.7 Hz, H-1), 7.14 (1*H*, d, J = 2.4 Hz, H-2), 6.93 (1*H*, dd, J = 8.7,2.4 Hz, H-3), 5.41 (1*H*, bs, -NH), 3.81 (3*H*, s, -OCH3); 13 C NMR (CDCl3, ppm): 166.2 (S-C=N), 145.1 (C-9), 138.6 (C-6), 126.5 (C-8), 124.6 (C-4), 121.4 (C-5), 116.5 (C-7), 56.8 (-OCH3); EIMS (70 eV): m/*z* (%); [*M*+.] 180 (51%); Anal. Calcd. for C8H8N2OS; C, 52.33; H, 4.44; N, 15.55; S, 17.77. Found: C, 52.26; H, 4.35; N, 15.37; S, 17.61..

### Refinement

Hydrogen atoms were clearly identified in difference syntheses, and were refined at idealized positions [C—N = 0.88 Å; C—H(aromatic) = 0.93 Å and C—H(methyl) = 0.96 Å], riding on the nitrogen or carbon atoms with isotropic displacement parameters *U*_iso_(H) = 1.2*U*_eq_(N, C_ar_) or 1.5*U*_eq_(C_methyl_). The hydrogen atoms were allowed to rotate but not to tip.

### Crystal data

**Table:**

C_8_H_8_N_2_OS	*D*_x_ = 1.425 Mg m^−^^3^
*M**_r_* = 180.23	Melting point = 418–420 K
Orthorhombic, *P**b**c**a*	Mo *K*α radiation, λ = 0.71073 Å
Hall symbol: -P 2ac 2ab	Cell parameters from 3739 reflections
*a* = 15.060 (2) Å	θ = 2.5–28.3°
*b* = 6.6997 (11) Å	µ = 0.33 mm^−^^1^
*c* = 16.649 (3) Å	*T* = 130 K
*V* = 1679.8 (5) Å^3^	Prism, colourless
*Z* = 8	0.47 × 0.23 × 0.14 mm
*F*(000) = 752	

### Data collection

**Table:**

Bruker SMART APEX CCD diffractometer	2010 independent reflections
Radiation source: sealed tube	1771 reflections with *I* > 2σ(*I*)
Graphite monochromator	*R*_int_ = 0.029
φ and ω scans	θ_max_ = 27.9°, θ_min_ = 2.5°
Absorption correction: multi-scan (*SADABS*; Sheldrick, 2004)	*h* = −19→19
*T*_min_ = 0.859, *T*_max_ = 0.955	*k* = −8→8
14745 measured reflections	*l* = −21→20

### Refinement

**Table:**

Refinement on *F*^2^	Primary atom site location: structure-invariant direct methods
Least-squares matrix: full	Secondary atom site location: difference Fourier map
*R*[*F*^2^ > 2σ(*F*^2^)] = 0.033	Hydrogen site location: difference Fourier map
*wR*(*F*^2^) = 0.094	H-atom parameters constrained
*S* = 1.06	*w* = 1/[σ^2^(*F*_o_^2^) + (0.0541*P*)^2^ + 0.5583*P*] where *P* = (*F*_o_^2^ + 2*F*_c_^2^)/3
2010 reflections	(Δ/σ)_max_ = 0.001
110 parameters	Δρ_max_ = 0.40 e Å^−^^3^
0 restraints	Δρ_min_ = −0.20 e Å^−^^3^

### Special details

**Table:**

Geometry. All e.s.d.'s (except the e.s.d. in the dihedral angle between two l.s. planes) are estimated using the full covariance matrix. The cell e.s.d.'s are taken into account individually in the estimation of e.s.d.'s in distances, angles and torsion angles; correlations between e.s.d.'s in cell parameters are only used when they are defined by crystal symmetry. An approximate (isotropic) treatment of cell e.s.d.'s is used for estimating e.s.d.'s involving l.s. planes.
Refinement. Refinement of *F*^2^ against ALL reflections. The weighted *R*-factor *wR* and goodness of fit *S* are based on *F*^2^, conventional *R*-factors *R* are based on *F*, with *F* set to zero for negative *F*^2^. The threshold expression of *F*^2^ > σ(*F*^2^) is used only for calculating *R*-factors(gt) *etc*. and is not relevant to the choice of reflections for refinement. *R*-factors based on *F*^2^ are statistically about twice as large as those based on *F*, and *R*- factors based on ALL data will be even larger.

### Fractional atomic coordinates and isotropic or equivalent isotropic displacement parameters (Å^2^)

**Table:**

	*x*	*y*	*z*	*U*_iso_*/*U*_eq_	
S1	0.67080 (2)	0.21698 (5)	0.37026 (2)	0.02447 (13)	
O1	0.68079 (6)	0.55013 (14)	0.09070 (6)	0.0234 (2)	
N1	0.56180 (7)	0.50795 (17)	0.40573 (6)	0.0217 (2)	
N2	0.58824 (8)	0.28778 (19)	0.51177 (7)	0.0292 (3)	
H2A	0.5525	0.3536	0.5442	0.035*	
H2B	0.6160	0.1805	0.5290	0.035*	
C1	0.60027 (9)	0.3510 (2)	0.43608 (8)	0.0220 (3)	
C2	0.58749 (8)	0.53507 (19)	0.32593 (8)	0.0191 (3)	
C3	0.56017 (9)	0.6894 (2)	0.27627 (8)	0.0225 (3)	
H3A	0.5211	0.7892	0.2962	0.027*	
C4	0.58993 (9)	0.6982 (2)	0.19719 (8)	0.0220 (3)	
H4A	0.5710	0.8036	0.1630	0.026*	
C5	0.64762 (8)	0.55186 (19)	0.16817 (8)	0.0193 (3)	
C6	0.67681 (8)	0.3964 (2)	0.21681 (8)	0.0209 (3)	
H6A	0.7162	0.2973	0.1968	0.025*	
C7	0.64648 (8)	0.39071 (19)	0.29540 (8)	0.0193 (3)	
C8	0.65274 (10)	0.7074 (2)	0.03804 (9)	0.0281 (3)	
H8A	0.5878	0.7068	0.0340	0.042*	
H8B	0.6786	0.6867	−0.0153	0.042*	
H8C	0.6726	0.8360	0.0595	0.042*	

### Atomic displacement parameters (Å^2^)

**Table:**

	*U*^11^	*U*^22^	*U*^33^	*U*^12^	*U*^13^	*U*^23^
S1	0.0272 (2)	0.0253 (2)	0.0208 (2)	0.00985 (13)	0.00107 (13)	0.00191 (12)
O1	0.0249 (5)	0.0256 (5)	0.0195 (5)	0.0008 (4)	0.0041 (4)	0.0015 (4)
N1	0.0242 (5)	0.0232 (5)	0.0177 (5)	0.0041 (4)	−0.0009 (4)	−0.0017 (4)
N2	0.0358 (7)	0.0314 (7)	0.0203 (6)	0.0133 (5)	0.0027 (5)	0.0039 (5)
C1	0.0220 (6)	0.0253 (7)	0.0187 (6)	0.0030 (5)	−0.0011 (5)	−0.0028 (5)
C2	0.0178 (6)	0.0215 (6)	0.0180 (6)	0.0010 (5)	−0.0013 (5)	−0.0027 (5)
C3	0.0230 (6)	0.0224 (6)	0.0223 (6)	0.0058 (5)	0.0005 (5)	−0.0014 (5)
C4	0.0220 (6)	0.0220 (6)	0.0220 (7)	0.0020 (5)	−0.0016 (5)	0.0017 (5)
C5	0.0177 (6)	0.0222 (6)	0.0181 (6)	−0.0034 (5)	0.0012 (5)	−0.0020 (5)
C6	0.0182 (6)	0.0217 (6)	0.0229 (7)	0.0026 (5)	0.0010 (5)	−0.0027 (5)
C7	0.0182 (6)	0.0196 (6)	0.0202 (6)	0.0018 (5)	−0.0027 (5)	−0.0012 (5)
C8	0.0339 (8)	0.0290 (8)	0.0213 (7)	−0.0006 (6)	0.0037 (6)	0.0052 (6)

### Geometric parameters (Å, º)

**Table:**

S1—C7	1.7443 (13)	C3—C4	1.3920 (19)
S1—C1	1.7705 (14)	C3—H3A	0.9500
O1—C5	1.3832 (16)	C4—C5	1.3962 (18)
O1—C8	1.4342 (17)	C4—H4A	0.9500
N1—C1	1.3028 (18)	C5—C6	1.3908 (18)
N1—C2	1.3956 (17)	C6—C7	1.3864 (18)
N2—C1	1.3417 (18)	C6—H6A	0.9500
N2—H2A	0.8800	C8—H8A	0.9800
N2—H2B	0.8800	C8—H8B	0.9800
C2—C3	1.3864 (18)	C8—H8C	0.9800
C2—C7	1.4083 (18)		
			
C7—S1—C1	88.73 (6)	C5—C4—H4A	120.1
C5—O1—C8	117.23 (10)	O1—C5—C6	114.99 (11)
C1—N1—C2	110.55 (11)	O1—C5—C4	123.59 (12)
C1—N2—H2A	120.0	C6—C5—C4	121.42 (12)
C1—N2—H2B	120.0	C7—C6—C5	117.76 (12)
H2A—N2—H2B	120.0	C7—C6—H6A	121.1
N1—C1—N2	123.98 (13)	C5—C6—H6A	121.1
N1—C1—S1	115.86 (10)	C6—C7—C2	121.99 (12)
N2—C1—S1	120.15 (11)	C6—C7—S1	128.57 (10)
C3—C2—N1	125.63 (12)	C2—C7—S1	109.44 (10)
C3—C2—C7	118.96 (12)	O1—C8—H8A	109.5
N1—C2—C7	115.41 (11)	O1—C8—H8B	109.5
C2—C3—C4	120.01 (12)	H8A—C8—H8B	109.5
C2—C3—H3A	120.0	O1—C8—H8C	109.5
C4—C3—H3A	120.0	H8A—C8—H8C	109.5
C3—C4—C5	119.86 (12)	H8B—C8—H8C	109.5
C3—C4—H4A	120.1		
			
C2—N1—C1—N2	179.99 (13)	C3—C4—C5—C6	−0.3 (2)
C2—N1—C1—S1	−0.87 (15)	O1—C5—C6—C7	179.48 (11)
C7—S1—C1—N1	0.50 (11)	C4—C5—C6—C7	0.19 (19)
C7—S1—C1—N2	179.67 (12)	C5—C6—C7—C2	0.6 (2)
C1—N1—C2—C3	−178.88 (13)	C5—C6—C7—S1	−179.95 (10)
C1—N1—C2—C7	0.90 (17)	C3—C2—C7—C6	−1.2 (2)
N1—C2—C3—C4	−179.23 (12)	N1—C2—C7—C6	179.05 (12)
C7—C2—C3—C4	1.0 (2)	C3—C2—C7—S1	179.27 (10)
C2—C3—C4—C5	−0.3 (2)	N1—C2—C7—S1	−0.53 (15)
C8—O1—C5—C6	180.00 (11)	C1—S1—C7—C6	−179.51 (13)
C8—O1—C5—C4	−0.72 (18)	C1—S1—C7—C2	0.04 (10)
C3—C4—C5—O1	−179.55 (12)		

### Hydrogen-bond geometry (Å, º)

**Table:**

*D*—H···*A*	*D*—H	H···*A*	*D*···*A*	*D*—H···*A*
N2—H2*A*···N1^i^	0.88	2.13	2.9774 (16)	163
N2—H2*B*···O1^ii^	0.88	2.10	2.9655 (16)	170

Symmetry codes: (i) −*x*+1, −*y*+1, −*z*+1; (ii) *x*, −*y*+1/2, *z*+1/2.
